# Psychometric validation of the Spanish alopecia areata–life impact questionnaire

**DOI:** 10.3389/fmed.2025.1706110

**Published:** 2025-11-13

**Authors:** Daniel Muñoz-Barba, Carmen García-Moronta, Miguel Limeres-De la Rosa, Manuel Sánchez-Díaz, Salvador Arias-Santiago

**Affiliations:** 1 Dermatology Unit, Hospital Universitario Virgen de las Nieves, Granada, Spain; 2 Dermatology Department, School of Medicine, University of Granada, Granada, Spain; 3 Institute of Biosanitary Research IBS, Granada, Spain; 4 Trichology Clinic, Hospital Universitario Virgen de las Nieves, Granada, Spain

**Keywords:** alopecia areata, quality of life, questionnaire validation, psychodermatology, trichology

## Abstract

**Introduction:**

Alopecia areata (AA) is a chronic immune-mediated disorder associated with substantial psychosocial burden. Generic dermatology instruments, such as the DLQI, may fail to capture AA-specific concerns, prompting the development of targeted questionnaires like the AA-QLI. However, existing tools show methodological limitations and inconsistent use. This study aimed to validate the Spanish Alopecia Areata–Life Impact Questionnaire (SAALIQ) by assessing its reliability, validity, and responsiveness, providing a robust instrument for evaluating the quality-of-life (QoL) impact of AA in Spanish-speaking patients.

**Methods:**

A single-center cross-sectional study was conducted between June 2024 and June 2025. Adults with AA, recruited sequentially regardless of disease severity, duration, or prior treatments, were included. QoL was assessed using generic and disease-specific questionnaires, including the SAALIQ, developed in collaboration with the Alopecia Association of the Community of Madrid. Psychometric validation included assessment of internal consistency, test–retest reliability, and convergent validity. Receiver-operating characteristic (ROC) analyses were performed to determine cut-off points.

**Results:**

A total of 85 patients with AA were included (72.94% women, 27.06% men) with a mean age of 37.55 years. The mean disease duration was 10.12 years, and the mean baseline SALT score was 36.52%. Cronbach’s *α* ranged from 0.73 to 0.80 across emotional, functional, and social domains. Intraclass correlation coefficient (ICC) for the total score was 0.947. Convergent validity was confirmed by moderate-to-strong correlations with DLQI, Hospital Anxiety and Depression Scale (HADS-A, HADS-D), and a strong correlation with the AA-QLI (*r* = 0.603; *ρ* = 0.714; *p* < 0.0001). Greater SAALIQ scores were observed in women, in patients with AA totalis (AAT) or multiple patch involvement, and in those with longer disease duration (*p* < 0.05). ROC analysis identified SAALIQ cut-offs of <20 for absent/mild, ≥20 for moderate, and >26 for severe QoL impairment.

**Conclusion:**

The SAALIQ is a disease-specific and culturally adapted tool that enables accurate measurement of the QoL impact of AA in Spanish-speaking patients. Its psychometric performance, validated cut-off points, and ability to capture domains overlooked by generic measures make it an essential resource for patient-centered care, clinical trials, and international research. Further studies are warranted to confirm the generalizability of our findings.

## Introduction

Alopecia areata (AA) is a chronic, immune-mediated disorder characterized by non-scarring hair loss that can progress to complete scalp or total body hair loss (1). Although AA is not physically disabling, its unpredictable course, visible nature, and potential for long-term persistence can exert a substantial psychosocial impact. Patients frequently experience emotional distress, anxiety, depressive symptoms, and reduced self-esteem, which may lead to social withdrawal and impaired quality of life (QoL) (2–4).

Assessment of the health-related quality of life (HRQoL) impact in AA has traditionally relied on generic dermatology instruments, such as the Dermatology Life Quality Index (DLQI) (5). While widely used, these measures may not adequately capture the disease-specific domains relevant to AA, including the psychological consequences of unpredictable relapses, concerns about public perception, and the functional impact of hair loss in daily activities. To address this gap, several disease-specific tools have been developed (6), including the Alopecia Areata Quality of Life Index (AA-QLI) (7).

Although these instruments have demonstrated acceptable psychometric performance, several methodological limitations remain, particularly the lack of patient participation in their development. Furthermore, their use has not been consistent across AA studies, which raises concerns regarding reproducibility. This inconsistency may be partly due to their considerable length or interpretative complexity, which often hinders both completion by patients and integration into routine clinical practice.

The objective of the present study was to validate the Spanish Alopecia Areata–Life Impact Questionnaire (SAALIQ) Questionnaire for use in Spanish-speaking patients with AA. The validation process evaluated internal consistency, test–retest reliability, construct validity, and responsiveness to clinical change, with comparisons to established measures such as the DLQI and AA-QLI. A robust, validated instrument is expected to provide clinicians and researchers with a reliable tool for assessing the specific QoL impact of AA in Spanish-speaking populations.

## Methods

### Study design

A review of existing AA-specific QoL measures was conducted (8). This was followed by semi-structured interviews with patient focus groups, members of the Community of Madrid Alopecia Association, and dermatologists and psychologists with expertise in the management of AA, in order to identify relevant thematic domains. Based on these findings, a preliminary version of the questionnaire was drafted with the objective of generating clear, relevant, and easily understood items. The previous expert panel subsequently reviewed each item for relevance, clarity, and representativeness to ensure content validity and conceptual coverage. The questionnaire was initially administered to a sample of 20 patients who were not included in the study. Based on their feedback, all 10 items were retained, although some questions were reformulated to enhance comprehension and minimize the risk of misinterpretation. After that, a single-center cross-sectional study was conducted including patients with AA attending the Dermatology Department in a third-level center between June 2024 and June 2025. Sequential sampling was applied. After providing written informed consent, patients were invited to share their clinical information and complete a series of questionnaires designed to assess the impact of AA on QoL.

### Inclusion criteria

The inclusion criteria were: (a) patients with a clinical diagnosis of AA, regardless of disease severity, duration and previous or current treatments; (b) being diagnosed with AA for almost 6 months; (c) age 18 years or older; (d) informed consent to be included in the study; (e) understanding of Spanish language so as to make it possible to complete the questionnaires.

### Exclusion criteria

The exclusion criteria were: (a) patient’s refusal to participate in the study; (b) patients who had any other major disease that may have impacted their mood or QoL.

### Ethics

The current study was approved by the Research Ethics Committee of Granada and was performed in accordance with the principles of the Declaration of Helsinki.

### Variables

Main Variables: The study focused on variables related to QoL and mood disorders. Data were collected using a set of questionnaires, classified into two categories: instruments specifically designed for patients with AA, and non-disease-specific instruments:

- Spanish Alopecia Areata–Life Impact Questionnaire (SAALIQ): It includes 10 questions referring to the 8 weeks prior to the medical consultation. Items 1 to 4 were related to emotional domain; items 5 to 7 were questions about functional domain; and items 8–10 questions were related to social domain. A Likert-type response format was employed, with a 4-point scale where 1 = “No,” 2 = “Yes, a little,” 3 = “Yes, quite a lot,” and 4 = “Yes, very much.” The total score ranges from 10 to 40, with higher scores indicating a greater negative impact (40 = worst QoL) and lower scores reflecting less impairment (10 = best QoL). The questionnaire was refined following interviews with 15 patients. Based on their initial feedback, several items were reworded to enhance clarity and ensure comprehension. The final version of the questionnaire was then administered to all study participants (details can be seen in Supplementary material).
- Alopecia Areata Quality of Life Index (AA-QLI): It is a disease-specific instrument designed to assess the QoL in patients with AA. It measures the psychosocial and functional impact of the condition across domains such as self-perception, emotional well-being, and daily activities. The questionnaire comprises 21 items, each scored on a Likert-type scale, with higher scores reflecting greater impairment. Domain and total scores are calculated by summing item responses (7).
- Dermatology Life Quality Index (DLQI): This instrument assesses overall dermatological QoL in individuals aged 16 years and older. It contains 10 items, each scored on a 4-point Likert scale from 0 (no impact) to 3 (maximum impact). Responses are based on the patient’s experiences during the previous week (5, 9).
- World Health Organization-Five Well-Being Index (WHO-5): This brief questionnaire evaluates mental well-being over the preceding 2 weeks. It comprises five items, each scored from 0 to 5 on a Likert scale, with higher values reflecting better well-being. Item scores are summed, multiplied by four, and expressed on a scale from 0 to 100. Scores below 50 suggest reduced mental well-being (10).
- Hospital Anxiety and Depression Scale (HADS): This validated tool consists of 14 items, each rated on a Likert scale. It includes two subscales: odd-numbered items measure anxiety and even-numbered items assess depression. Scores of 8 or higher on either subscale indicate probable anxiety or depression, respectively (11, 12).

Secondary Variables: Sociodemographic variables were collected through a multiple-choice section included at the beginning of the self-administered patient questionnaires, while clinical variables were obtained from the electronic medical records completed during the consultation. The severity of was assessed following Severity of Alopecia Tool (SALT) (13).

### Statistical analysis

The psychometric evaluation of the SAALIQ was performed in relation to the DLQI, HADS, and AA-QLI. Reliability was assessed through internal consistency, calculated using Cronbach’s *α* (considered acceptable when >0.7), and reproducibility, evaluated using the intraclass correlation coefficient (ICC; acceptable when >0.7). Test–retest reliability was determined by calculating the ICC for the total score. Convergent validity, defined as the degree to which measures of the same construct are related, was examined using Pearson’s correlation coefficient to assess associations between SAALIQ raw scores and those of the DLQI, HADS, and AA-QLI. Cut-off values for categorizing QoL impairment were established through receiver operating characteristic (ROC) curve analysis, using DLQI categories as references.

Continuous variables are reported as mean ± standard deviation (SD) and categorical variables as relative (absolute) frequencies. Normality of continuous data was tested with the Kolmogorov–Smirnov test. Mean differences in quantitative variables were assessed using Student’s t-test, and associations between qualitative variables were examined using the χ^2^ test. Statistical significance was set at *p* < 0.05 (two-tailed). Data analysis was performed using SPSS Statistics, version 24.0 (SPSS Inc., Chicago, IL, United States).

For sample size estimation, a minimum of four participants per variable was considered necessary for questionnaire validation (14). Given that the SAALIQ comprises 10 items, the target sample size was at least 40 participants.

## Results

### Sociodemographic and clinical characteristics of the sample

A total of 85 patients with AA were included in the study. The mean age was 37.55 years (SD 14.58), with a predominance of females (72.94%, *n* = 62) over males (27.06%, *n* = 23). Tobacco use was reported by 31.76% of patients. Most participants had completed higher education (62.35%), were employed (61.15%), and were either single (45.88%) or married (38.83%; details can be seen in Table 1).

**Table 1:** Socio-demographic and clinical features of patients with AA of the sample.

Socio-demographic features of the patients with AA (N = 85)			
Age (years)		37.55 (SD 14.58)	
Sex		Female 72.94% (62/85)	
		Male 27.06% (23/85)	
		F: M ratio 2.7 (62/23)	
Tobacco (%)		Smokers 31.76% (27/85)	
		Non-smokers 68.24% (58/85)	
Educational level		Non or basic studies 8.24% (7/85)	
		Secondary studies 29.41% (25/85)	
		University studies 62.35%(53/85)	
Occupation		Student 20.00% (17/85)	
		Employed 61.15% (52/85)	
		Unemployed 12.95% (11/85)	
		Retired 5.90% (5/85)	
Marital status		Single 45.88% (39/85)	
		Married 38.83% (33/85)	
		Divorced 15.29% (13/85)	
Clinical characteristics of the patients with AA (*N* = 85)			
Evolution time of the disease (years)	10.12 (SD 9.42)		
Age of debut (years)	27.42 (SD 14.08)		
Basal SALT score (%)	36.52% (SD 39.77)		
AA subtype	Multiple plaques AA (%)	56.47% (48/85)	
	AA Total or Universalis (%)	43.53% (37/85)	
Special locations	Ofiasic 12.94% (11/85)		
	Eyebrows/eyelashes 36.47% (31/85)		
	Beard 60.87% (14/23)		
	Body hair 24.71% (21/85)		
Previous treatments	Topical or oral minoxidil: 44.47% (38/85)	Topical corticosteroids: 31.77% (27/85)	Intralesional corticosteroids: 18.82% (16/85)
	Oral corticosteroids: 16.47% (14/85)	Baricitinib: 43.53% (37/85)	Ritlecitinib: 4.71% (4/85)

AA, Alopecia Areata; SALT, Severity of Alopecia Tool; SD, standard deviation.

The mean disease duration was 10.12 years (SD 9.42), with a mean age of onset of 27.42 years (SD 14.08). The mean baseline SALT score was 36.52% (SD 39.77). Multiple patch AA was present in 56.47% (*n* = 48) of patients, while 43.53% (*n* = 37) had total or universal AA (AAT or AAU). Special site involvement included ophiasis pattern (12.94%), eyebrows/eyelashes (36.47%), beard (60.87% of males), and body hair (24.71%). Regarding current treatments, 44.47% (*n* = 38) were receiving topical or oral minoxidil, 31.77% (*n* = 27) topical corticosteroids, 18.82% (*n* = 16) intralesional corticosteroids, and 16.47% (*n* = 14) oral corticosteroids. Systemic therapies included methotrexate in 2.23% (*n* = 2), baricitinib in 43.53% (*n* = 37), and ritlecitinib in 4.71% (*n* = 4; details can be seen Table 1).

### Impact of AA on QoL based on questionnaire scores

In the study population, the mean DLQI score was 5.49 ± 6.36, with 32.5% of patients reporting no impact on QoL, 31.25% mild impact, and 36.25% moderate-to-extreme impact. The mean HADS-A score was 9.16 ± 5.24, with 60% of participants presenting anxiety symptoms. The mean HADS-D score was 5.19 ± 4.33, with depressive symptoms identified in 28.75% of cases. The WHO-5 Well-Being Index mean score was 13.54 ± 6.08, with 42.5% of patients exhibiting low well-being. The mean SAALIQ score was 21.59 ± 6.78, with 61.25% of participants showing moderate-to-severe disease-specific quality-of-life impairment. When analyzing the SAALIQ by domains, the highest mean raw score corresponded to the emotional domain (10.64 ± 3.32), followed by the functional (7.11 ± 2.70) and social domains (5.21 ± 2.47). The mean original AA-QLI score was 49.73 ± 16.52. Scores were also standardized to a 0–100 scale to enable comparability in subsequent analyses (details can be seen in Table 2).

**Table 2:** Descriptive statistics for quality-of-life and psychological well-being measures in the study population.

Quality-of-life and psychological well-being scores in patients with AA (*N* = 85)					
DLQI score	5.49 ± 6.36	DLQI score_100_	18.29 ± 21.20	DLQI categories	Absent: 32.50%
					Mild: 31.25%
					Moderate: 22.50%
					Severe: 10.00%
					Extreme: 3.75%
HADS-A score	9.16 ± 5.24	HADS-A score_100_	43.63 ± 24.95	HADS-A (%)	Non-anxiety: 40.00%
					Anxiety: 60.00%
HADS-D score	5.19 ± 4.33	HADS-D score_100_	24.70 ± 20.63	HADS-D (%)	Non-depression: 71.25%
					Depression: 28.75%
WHO-5 score	13.54 ± 6.08	WHO-5 score_100_	45.15 ± 26.01	WHO-5 impairment (%)	Absent: 57.50%
					Low: 45.50
SAALIQ score	21.59 ± 6.78	SAALIQ score_100_	53.97 ± 16.95	SAALIQ impairment	Absent or mild: 38.75%
					Moderate: 33.75%
					Severe: 25.00%
SAALIQ domains score	Emotional 10. 64 ± 3.32		SAALIQ domains score_100_	Emotional 66.53 ± 20.79	
				Functional 59.32 ± 22.49	
	Functional 7.11 ± 2.70				
	Social 5.21 ± 2.47			Social 43.42 ± 20.58	
AA-QLI score	49.73 ± 16.52		AA-QLI score_100_	59.20 ± 19.67	

Data are expressed as mean ± standard deviation (SD) for raw and transformed (0–100) scores, and as absolute percentages for categorical classifications. DLQI, Dermatology Life Quality Index; HADS-A, Hospital Anxiety and Depression Scale–Anxiety subscale; HADS-D, Hospital Anxiety and Depression Scale–Depression subscale; WHO-5, World Health Organization Well-Being Index; SAALIQ, Alopecia Areata–Life Impact Questionnaire Spanish version; AA-QLI, Alopecia Areata-Quality of Life Index.

### Psychometric validation of the SAALIQ

Reliability was evaluated through internal consistency and temporal stability. Internal consistency, assessed using Cronbach’s *α* for each of the three domains, yielded values of 0.7785 for the emotional domain, 0.7294 for the functional domain, and 0.8023 for the social domain. The most internally consistent items within each domain were items 1, 6, and 9, respectively. Temporal stability was examined in a subsample of 25 patients with AA using ICC between test and retest administrations, with an interval of 30 days. The ICC for the total score was 0.9475, indicating excellent reproducibility, with no significant changes observed that could be attributed to ongoing treatment during this period (details can be seen in Table 3).

**Table 3:** Summary of the psychometric properties of the SAALIQ.

Psychometric property and corresponding values for each domain and total score				
Psychometric property	Emotional domain	Functional domain	Social domain	Total score/other
Internal consistency (Cronbach’s α)	0.7785	0.7294	0.8023	
Most consistent item	Item 1	Item 6	Item 9	
Test–retest reliability (ICC, total score)				0.9475
Convergent validity (Pearson’s r)	DLQI: 0.3239	HADS-A: 0.2798	HADS-D: 0.3147	AA-LIQ: 0.6027
Convergent validity (Spearman’s ρ)	DLQI: 0.4622	HADS-A: 0.3523	HADS-D: 0.3862	AA-LIQ: 0.7136

ICC, intraclass correlation coefficient; DLQI, Dermatology Life Quality Index; HADS-A, Hospital Anxiety and Depression Scale–Anxiety subscale; HADS-D, Hospital Anxiety and Depression Scale–Depression subscale; WHO-5, World Health Organization Well-Being Index; AA-QLI, Alopecia Areata-Quality of Life Index.

Validity was assessed through convergent and criterion-related approaches. Convergent validity was determined by comparing SAALIQ scores with those from other instruments assessing the impact of AA, standardized to a 0–100 scale. In parametric analyses, the SAALIQ showed moderate positive correlations with the DLQI (*r* = 0.3239), the HADS-A anxiety subscale (*r* = 0.2798), and the HADS-D depression subscale (*r* = 0.3147), and a strong correlation with the AA-LIQ (*r* = 0.6027). Non-parametric Spearman analyses confirmed these associations, with significant correlations observed for the DLQI (*ρ* = 0.4622), HADS-A (ρ = 0.3523), HADS-D (ρ = 0.3862), and AA-LIQ (ρ = 0.7136; all *p* values < 0.0001; details can be seen in Table 3).

Criterion validity was explored by assessing associations between SAALIQ scores and sociodemographic or clinical variables. Univariate analysis showed significantly higher mean scores in females than in males (*p* = 0.02) and in patients with multiple-type and AAT compared with single-type AA (23.07 ± 0.69 vs. 18.19 ± 1.15). Longer disease duration was also positively associated with higher scores (coefficient = 0.13 ± 0.06; *p* = 0.04). No significant associations were found with age, age at onset, baseline SALT score, overall disease severity, or special site involvement (all *p* > 0.05; details can be seen in Table 4).

**Table 4:** Univariate analysis to explore association between sociodemographic and clinical characteristics and SAALIQ scores (*N* = 85).

	SAALIQ score mean ± SE (*N* = 85)	*p* value
Gender	Female: 22.37 ± 0.61	0.02
	Male: 19.37 ± 1.08	
Age (years)	−0.02 ± 0.04	0.54
Age at onset (years)	−0.04 ± 0.04	0.42
Disease duration (years)	0.13 ± 0.06	0.04
Basal SALT score (%)	−0.01 ± 0.01	0.39
Severe AA	Yes: 21.04 ± 0.99	0.47
	No: 21.89 ± 0.64	
Type of AA	Single: 18.19 ± 1.15	0.01
	Multiple: 23.07 ± 0.69	
	Total: 23.67 ± 2.66	
	Universal: 20.67 ± 1.19	
Special location involvement	Yes: 21.42 ± 0.66	0.57
	No: 22.07 ± 0.92	
Treatment	Topical: 21.56	0.96
	Systemic: 21.61	

AA, Alopecia Areata; SAL, Severity of Alopecia Tool.

### Determination of SAALIQ cut-off points for categorizing QoL impairment

**Figure 1:**
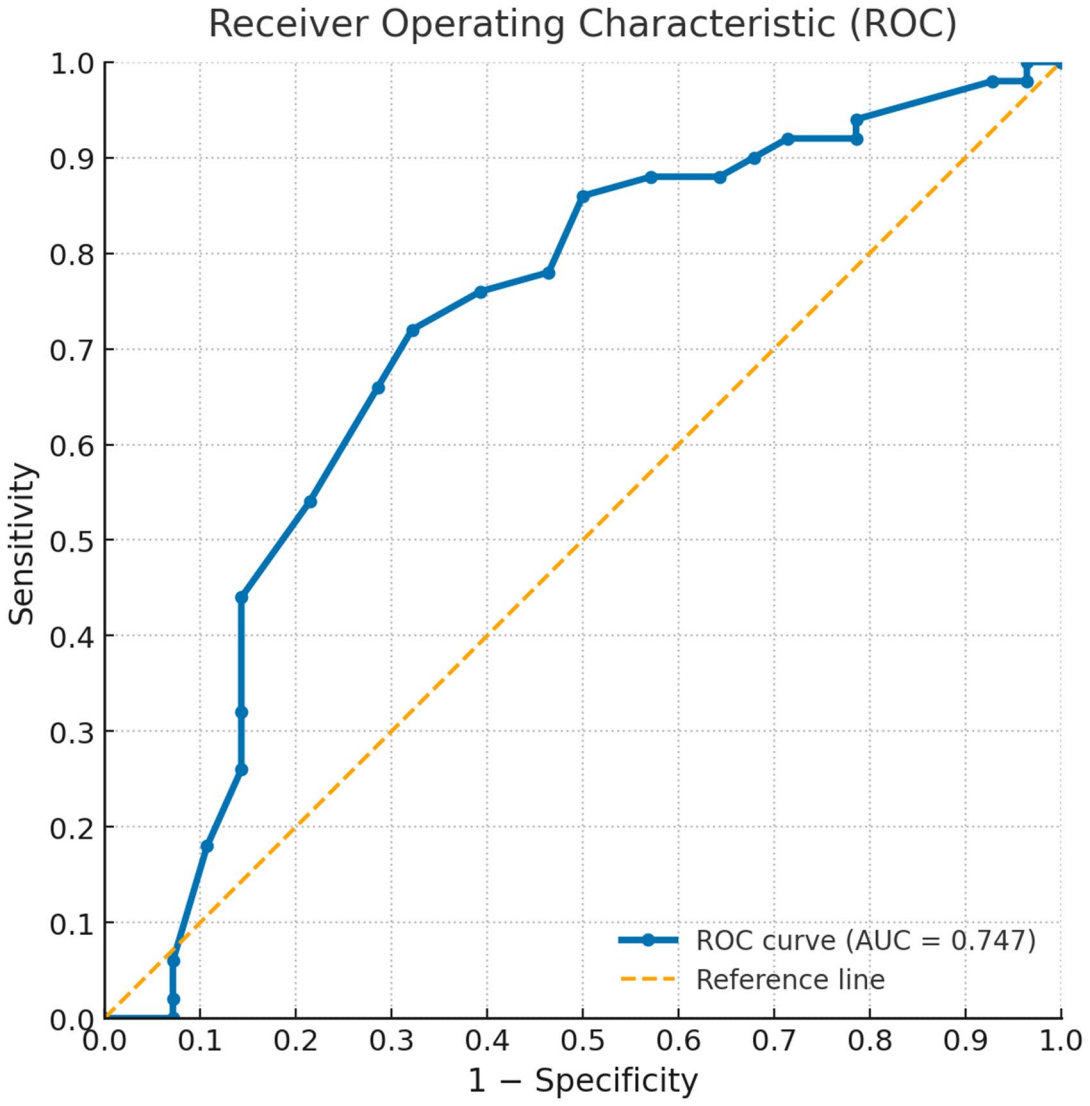
ROC curve for the SAALIQ questionnaire in discriminating patients with absent or mild QoL impairment (SAALIQ < 20), using the dichotomised DLQI as the reference standard. The area under the curve (AUC) was 0.747.

**Figure 2:**
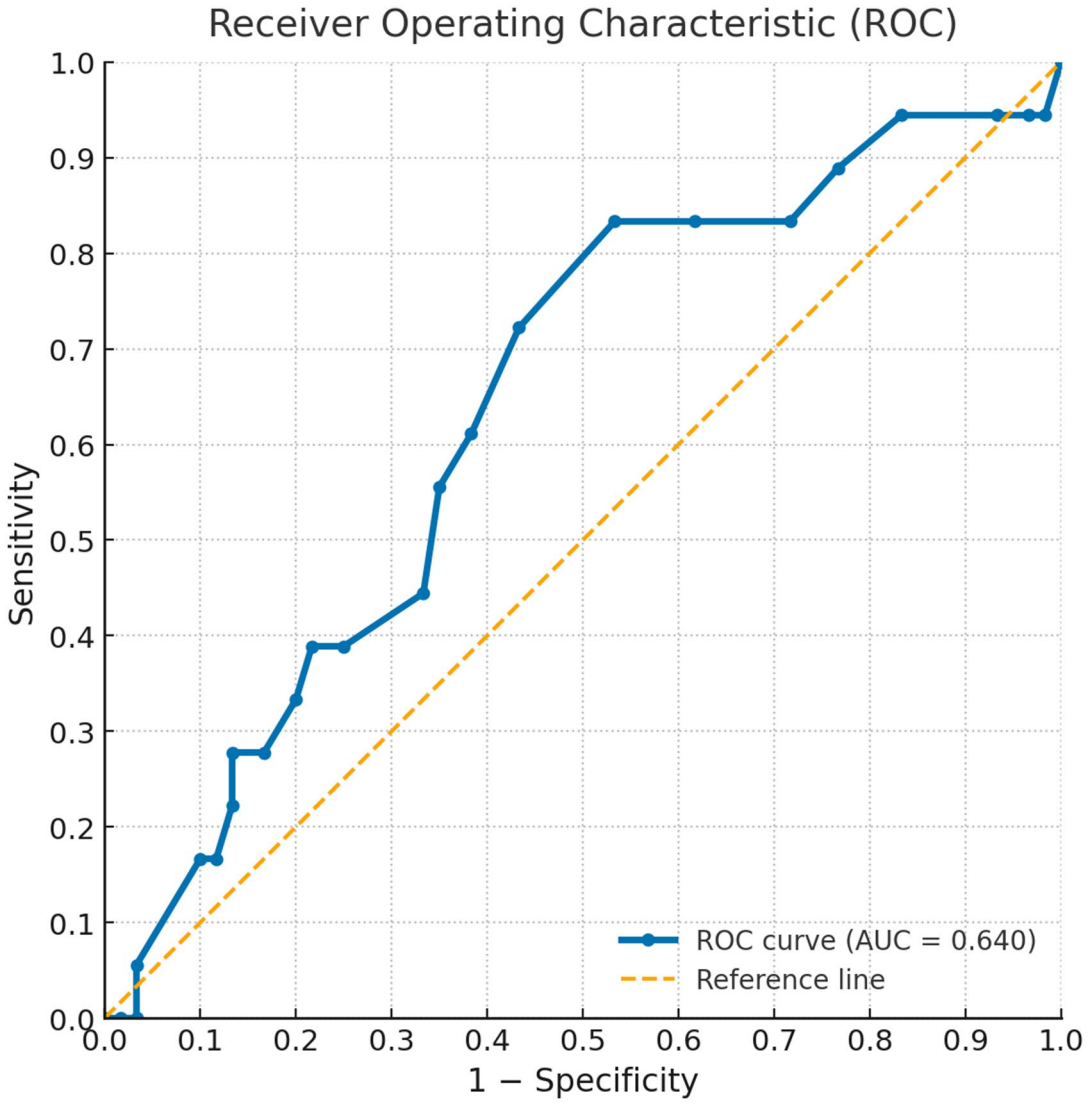
ROC curve for the SAALIQ questionnaire in discriminating patients with moderate QoL impairment (SAALIQ ≥ 20), using the dichotomised DLQI as the reference standard. The area under the curve (AUC) was 0.640.

**Figure 3:**
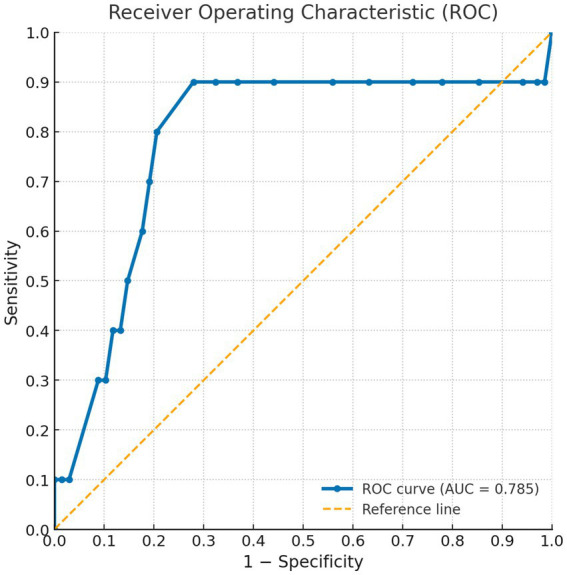
ROC curve for the SAALIQ questionnaire in discriminating patients with severe QoL impairment (SAALIQ > 26), using the dichotomised DLQI as the reference standard. The area under the curve (AUC) was 0.785.

Cut-off point was delimited by comparing the values for the SAALIQ and DLQI. The DLQI was used because it yielded the acceptable correlation values. The ROC curve analysis showed that values < 20 in the SAALIQ corresponded to patients with absent or mild QoL impairment measured by DLQI, with a sensitivity of 66% and a specificity of 78.57% (AUC = 0.746, *p* < 0.001). The ROC curve analysis showed that values ≥ 20 in the SAALIQ corresponded to patients with moderate QoL impairment measured by DLQI, with a sensitivity of 83.33% and a specificity of 46.67% (AUC = 0.640, *p* < 0.001). The ROC curve analysis showed that values > 26 in the SAALIQ corresponded to patients with severe QoL impairment measured by DLQI, with a sensitivity of 70% and a specificity of 80.88% (AUC = 0.785, *p* < 0.001; details can be seen in Figures 1–3).

## Discussion

The results of this study confirm that AA has a significant impact on patients’ QoL, with over one-third of the sample experiencing moderate-to-extreme impairment according to the DLQI and more than 60% presenting moderate-to-severe disease-specific impairment as measured by the SAALIQ. This is likely attributable to the greater ability of the SAALIQ in detecting QoL impairment directly related to AA. Furthermore, these findings are consistent with previous literature reporting a substantial psychosocial burden in AA (15–17), often comparable to that observed in chronic inflammatory dermatoses such as psoriasis (18) or atopic dermatitis (19).

Compared with generic dermatology instruments such as the DLQI, the SAALIQ also demonstrated greater specificity in capturing domains particularly relevant to AA, including the functional limitations of hair loss, social avoidance due to visible alopecia, and emotional distress related to disease unpredictability. Such disease-specific sensitivity is essential for accurately detecting changes in patient-reported outcomes, particularly in clinical trials or interventions targeting psychosocial health, and for avoiding the limitations inherent to non-specific measures.

Female patients reported significantly higher SAALIQ scores compared with males, consistent with prior literature suggesting that the psychosocial consequences of hair loss may be more pronounced in women due to societal and cultural perceptions of appearance (20–22). Similarly, individuals with more extensive clinical phenotypes—such as multiple-type AA and AAT—had markedly higher scores than those with single-plaque disease. However, no correlation was observed between SAALIQ scores and baseline SALT values (*p* > 0.10). This aligns with heterogeneous findings in the literature, where factors other than SALT, such as anxiety and depression, often emerge as stronger determinants of QoL than the extent of AA, underscoring the inconsistent relationship between objective severity and patient-reported impairment (23). Moreover, longer disease duration was positively associated with higher SAALIQ scores, indicating a cumulative psychosocial impact over time (24) as it occurs in other dermatosis (25, 26). We believe that this lifelong accumulated impact may play a more decisive role in determining the overall disease burden than the mere extent of AA.

The availability of a validated Spanish-language instrument such as the SAALIQ represents a significant advance for both clinical practice and research. Notable strengths include its development through close collaboration between AA specialists and patients, ensuring that it addresses the most relevant concerns and domains affected by the disease, thereby enhancing external validity. Furthermore, the inclusion of patients with mild disease and those undergoing a wide range of treatments increases the representativeness of the sample. In routine clinical practice, the SAALIQ can help dermatologists identify patients in need of psychological support and monitor changes over time, while in research settings it facilitates the collection of relevant and comparable data regarding QoL impairment. Moreover, it showed an adequate correlation with the previously validated AA-QLI (7), supporting its construct validity. Compared with the latter, the SAALIQ offers several advantages. Its shorter format, with only 10 items versus the 21 of the AA-QLI, enables faster completion and reduces respondent fatigue, as it can be completed in approximately half the time. In addition, its straightforward structure facilitates clearer interpretation by clinicians. Most importantly, the SAALIQ provides validated cut-off points to categorize the degree of QoL impairment, thereby offering a practical framework for therapeutic decision-making and ensuring comparability across studies. Finally, SAALIQ extends the recall period to the 8 weeks prior to the visit, encouraging deeper reflection on the long-term impact of the disease without incurring recall bias. Moreover, it has to be taken into account that other disease-specific questionnaires have been developed for other specific alopecias, such as scarring alopecias (27).

This study has certain limitations. The sample size, although adequate for psychometric validation, limits the ability to perform subgroup analyses. The single-center design may restrict the generalizability of the findings, and potential selection bias cannot be excluded, as participants were recruited from a tertiary dermatology clinic. Furthermore, the cross-sectional design precludes assessment of the instrument’s responsiveness to clinical change over time. Moreover, this questionnaire has been validated in a specific area of Spain, not in other Spanish-talking areas such as Latin America.

Future research should focus on longitudinal studies to confirm the sensitivity of the SAALIQ to changes in disease activity and treatment response. Its application in clinical trials could provide more accurate estimates of patient-reported outcomes and help define clinically meaningful changes in QoL scores. Additionally, cross-cultural adaptation and validation in other languages would allow for broader international comparability and facilitate multinational research collaborations.

## Conclusion

The SAALIQ is a disease-specific and culturally adapted tool that enables accurate measurement of the QoL impact of AA in Spanish-speaking patients. Its strong psychometric performance, validated cut-off points, and ability to capture domains overlooked by generic measures make it an essential resource for patient-centered care, clinical trials, and international research. Further studies are warranted to confirm the generalizability of our findings.

## Data Availability

The raw data supporting the conclusions of this article will be made available by the authors, without undue reservation.
