# Supplementary material for: Psychometric validation of the Spanish alopecia areata–life impact questionnaire
Source: Front Med (Lausanne). 2025 Nov 13;12:1706110. doi: 10.3389/fmed.2025.1706110 (PMC12657179; doi:10.3389/fmed.2025.1706110)
Supplement: Supplementary file 1 [file Table_1.docx]

Supplementary Material

**Supplementary Material 1.** Spanish Alopecia Areata–Life Impact Questionnaire (SAALIQ). The questionnaire comprises 10 items grouped into three domains: emotional (items 1–4), functional (items 5–7), and social (items 8–10). Each item is answered on a 4-point Likert scale ranging from 1 (“No”) to 4 (“Yes, very much”), with higher scores indicating greater quality-of-life impairment. The recall period covers the 8 weeks prior to the medical consultation.

**Spanish Alopecia Areata Life Impact Questionnaire (SAALIQ)**

Responda a las siguientes preguntas relacionadas sobre el posible impacto que la alopecia areata ha tenido en diferentes ámbitos de su vida durante **las últimas 8 semanas:**

**Dominio Emocional**:

Pregunta 1. ¿Considera que la alopecia que padece ha afectado negativamente a su estado de ánimo?

1. No.
2. Sí, un poco.
3. Sí, bastante.
4. Sí, mucho.

Pregunta 2. ¿Ha pensado que preferiría padecer un problema de salud interno (más importante, pero no visible, como la hipertensión o la diabetes) en lugar de la alopecia?

1. No.
2. Sí, un poco.
3. Sí, bastante.
4. Sí, mucho.

Pregunta 3. ¿Le preocupa la pérdida de las cejas o las pestañas?

1. No.
2. Sí, un poco.
3. Sí, bastante.
4. Sí, mucho.

Pregunta 4. ¿Piensa diariamente en el problema que padece?

1. No.
2. Sí, un poco.
3. Sí, bastante.
4. Sí, mucho.

**Dominio Funcional**:

Pregunta 5. ¿ Considera que hace uso de algún método para disimular su alopecia (peinado diferente, tatuaje de cejas, maquillaje de cejas, uso de pelucas, sistema capilar…) ?

1. No.
2. Sí, un poco.
3. Sí, bastante.
4. Sí, mucho.

Pregunta 6.¿Ha modificado sus rutinas de higiene diarias por miedo a empeorar la alopecia (frecuencia de lavado de cabello, uso de champú o acondicionador…) ?

1. No.
2. Sí, un poco.
3. Sí, bastante.
4. Sí, mucho.

Pregunta 7.¿Considera que dedica demasiado tiempo a la realización de tratamientos médicos para la alopecia que padece (aplicación de productos, toma de medicación, citas médicas…)?

1. No.
2. Sí, un poco.
3. Sí, bastante.
4. Sí, mucho.

**Dominio Social**:

Pregunta 8.¿Considera que la alopecia interfiere o ha interferido negativamente en su vida laboral? (Obtención del puesto de trabajo deseado, pérdida de días de trabajo por bajas laborales, despidos porque la alopecia alteraba mi imagen personal…).

1. No.
2. Sí, un poco.
3. Sí, bastante.
4. Sí, mucho.

Pregunta 9.¿Considera que la alopecia interfiere o ha interferido negativamente en su vida sexual o sus relaciones de pareja?

1. No.
2. Sí, un poco.
3. Sí, bastante.
4. Sí, mucho.

Pregunta 10.¿Considera que la alopecia interfiere o ha interferido negativamente en la relación con sus amistades o sus relaciones sociales?

1. No.
2. Sí, un poco.
3. Sí, bastante.
4. Sí, mucho.
